# What Do You Know about Reproductive Medicine? – Results of a German Representative Survey

**DOI:** 10.1371/journal.pone.0050113

**Published:** 2012-12-03

**Authors:** Yve Stoebel-Richter, Kristina Geue, Ada Borkenhagen, Elmar Braehler, Kerstin Weidner

**Affiliations:** 1 Department of Medical Psychology and Medical Sociology, University of Leipzig, Leipzig, Germany; 2 Clinic and Polyclinic for Psychotherapy and Psychosomatics, Medical Faculty Carl Gustav Carus, Dresden, Germany; University of Science and Technology of China, China

## Abstract

**Objective:**

The use of reproductive medical treatments has become increasingly routine in recent years. This paper reports on a study of how different aspects of modern reproductive medicine are perceived by the German population.

**Design:**

Findings from a nationally representative sample of 2110 men and women aged 18 to 50 are presented. Participants responded to a questionnaire seeking self-report information about attitudes and knowledge regarding different aspects of reproductive medicine.

**Results:**

The majority of respondents had already heard or read something about reproductive medicine; knowledge gaps were prevalent in men and individuals with lower levels of education. The decrease in female fertility usually was underestimated, whereas both the number of involuntarily childless couples and the success rate of reproductive medical treatment were overestimated. One-third of participants would make use of reproductive medicine to have their own child.

**Conclusion:**

This study revealed inadequacies in the knowledge of the German general population regarding reproductive medicine. Despite the low interest and poor knowledge of the topic, a broad acceptance of reproductive medical methods was reported. The results illustrate the need for adequate information transfer regarding female fertility as well as success rate and risks of reproductive medical interventions.

## Introduction

Since the first successful use of IVF in 1978 [1], assisted reproductive medicine has made great progress, has become routine in the health care system [2], and is constantly developing [3], [4], [5].

About 6–9% of all couples in central Europe remain involuntarily childless and desire treatment [6], [7]. Assessments of lifetime risk of infertility suggest that 20–30% of all couples will be affected by reduced fertility once in their life. In many cases, couples associate modern reproductive methods with a reliable possibility of achieving parenthood for almost an unlimited period of time [8], [9], [10]. In contrast, previous studies have reported success rates of IVF treatment of lower than 20% [3], [11], [12]. Many studies demonstrate a relationship between the age of the woman treated and treatment success of assisted reproductive medicine: the older the women at treatment time, the lower the chance of success [12], [13], [14]. The rapid decrease of gravidities in women older than 35 years who have IVF-treatment corresponds with the decline of the general pregnancy probability in middle-aged women. Against the background of the predominantly unsuccessful results of assisted reproduction methods, patient-centered treatment requires a careful consideration of risks and benefits of medical treatment as well as the risks and benefits of nonmedical alternatives like adoption or living without children [15], [16], [17], [18].

Several studies have examined knowledge and attitudes towards fertility or reproductive medicine in certain population groups. A fundamentally positive attitude towards parenthood was found by Lampic et al. [19] in a survey of 401 Swedish students. But half of the female students envisioned their first-time parenthood as occurring after age 35, unaware of their own decreasing fertility. Approximately half of the participants supposed the time of decline in fertility to begin some years later than in actuality. Findings of the study by Tough et al. [20] also found that women are not aware of risks associated with delayed parenthood. The authors stated that participants’ postponement of first-time parenthood is associated with insufficient knowledge about their own reproductive capability. A study by Quach et al. [4] found that 80% of 772 high school students underestimated the infertility rate, and nearly all of the respondents believed that infertility would be ‘curable’. Knowledge gaps were also reported by Rovei et al. [21] in a study of Italian students (N = 958) among whom this information deficit was uncorrelated with sex and type of education (humanities or science). Whereas the decline in female fertility was underestimated, the pregnancy rate after either sexual intercourse or IVF was overestimated. Rovei and collegues [21] recognized a better information level of people as an essential element in decision-making concerning when to start a family. The Finnish study by Heikkilä et al. [2] exploring the attitudes towards reproductive medicine of infertile women (N = 189) and breast-feeding mothers (N = 84) demonstrated similar attitudes in both groups. Infertile women, however, were even more liberal and favored a popularization of reproductive medicine.

Only half of 205 surveyed American physicians (general practitioners, internists, gynecologists) associated infertility with women aged 35 and older [22]. The majority of physicians supposed European Americans with high income to be at the highest risk for infertility (85%). In fact, this group makes the most frequent use of reproductive medicine, which likely account for this, selective perception on the part of these professionals. Ceballo et al. [22] required a better education of non-experts regarding infertility treatment. Papaharitou et al. [23] compared attitudes of midwives with the general population and found no differences between the groups. But there was an effect of age insofar as older respondents favored stronger regulation of reproductive medicine.

The aforementioned studies indicate knowledge gaps or false beliefs about fertility, infertility, and reproductive prospects among both medical professionals and nonprofessionals, while the latter consisting predominantly of young student samples. As an essential criterion for deciding on political questions, Kalfouglou and colleagues [15] highlighted knowledge of the attitudes of the general population (e.g. a public information campaign). For reproductive endocrinologists per se, knowledge about public opinion leads to a better understanding of couples being treated for infertility.

Our present study examined fertility-related knowledge and acceptance of modern assisted reproductive techniques using a large nationally representative sample of German adults aged between 18 and 50 years. Furthermore, the study sought to clarify how gender and level of education may influence these findings.

## Materials and Methods

### Ethics Statement

This representative survey was assigned by the University of Leipzig and carried out by USUMA (Berlin), a market analysing and opinion research institute. All participants were visited in person, informed about the study procedures by a trained research assistant, and signed an informed consent form prior to assessment. All interviewers/researchers involved were aware of the responsibility for confidentiality in respect to participants’ records. The data used were de-identified. The study adhered to the ethical guidelines of the ICC/ESOMAR International Code of Marketing and Social Research Practice. The present study posed a low risk to the participants. Ethical approval was required and obtained from the Ethic Committee by Leipzig University’s Institutional Review Board.

### Subjects

Respondents were randomly selected according to the random-route-procedure which first provided a target–home and after that a target–person in every home. The sample was weighted regarding sex and age to reflect the characteristics of the German population. The selected individuals were personally approached by the interviewer who collected the sociodemographic information face-to-face. The self-report attitude questionnaire was filled out by the respondents themselves in the presence of the interviewer. With a response rate of 55%, 2110 interviews were completed by persons aged 18 to 50 years.

### Instruments

Participants received a questionnaire which was designed to collect self-report information about attitudes and knowledge of different aspects of reproductive medicine. Several items were developed for this study. All instruments were pretested in several ways.

Participants were asked about various aspects of reproductive medicine in general. Knowledge-based questions about female fertility, the success rate of IVF, and childlessness in Germany were presented with multiple-choice or estimation response options. To find out about attitudes towards ethically controversial aspects of reproductive medicine, a set of statements including pros and cons was given. These items were related to the pushing of the biological age limit, to the advantages of remaining childless, and to moral conflicts. A five-point Likert scale was used to measure of degree of agreement with each statement. At the end of the questionnaire respondents were asked to evaluate the pros and cons of reproductive medicine in general using a six-point scale.

### Statistical Analysis

Data were analysed with descriptive as well as inferential statistical procedures. Significance levels of differences between frequencies were assessed through Chi^2^-analysis.

## Results

### Socio-demographic Information

The sample consisted of 929 males and 1181 females with an average age of 35.8 years (SD 9.1). More than half of all respondents (64.7%) were in a committed relationship and had at least one child (60%). A small number of participants 1.3% had themselves had infertility treatment (IVF 56%, hormonal treatment 30%). More information is given in Table 1.

**Table 1:** Socio-demographic characteristics of the sample.

		Total (N = 2110)	Female (N = 1181)	Male (N = 929)
**Age groups**	18–25 years	367 (17.4%)	193 (16.3%)	174 (18.7%)
	26–35 years	609 (28.9%)	343 (29.0%)	266 (28.6%)
	36–45 years	754 (35.7%)	433 (36.7%)	321 (34.1%)
	>45 years	380 (18.0%)	212 (18.0%)	168 (18.1%)
**Marital status**	Married, living together	1138 (53.9%)	680 (57.6%)	458 (49.3%)
	Married, separated	29 (1.4%)	16 (1.4%)	13 (1.4%)
	Single	712 (33.7%)	328 (27.8%)	384 (41.3%)
	Divorced	194 (9.2%)	127 (10.7%)	67 (7.2%)
	Widowed	37 (1.8%)	30 (2.5%)	7 (0.8%)
**Partnership**	yes	1365 (64.7%)	798 (67.6%)	567 (61.0%)
**Education degree**				
***low***	Less than 10th grade	644 (30.5%)	346 (29.3%)	298 (32.0%)
	10th grade	974 (46.2%)	589 (49.9%)	385 (41.5%)
***high***	Student	20 (0.9%)	6 (0.5%)	14 (1.5%)
	High school	321 (15.2%)	186 (15.7%)	135 (14.6%)
	College/university degree	151 (7.2%)	54 (4.6%)	97 (10.4%)
**Religion**	yes	1576 (74.7%)	908 (77.0%)	668 (71.9%)
**Children**	yes	1275 (60.4%)	803 (68.0%)	472 (50.8%)
**Used Assisted Reproductive Medicine**	yes	27 (1.3%)	24 (2.0%)	3 (0.3%)

### Participants’ Desire for Having Children

**Figure 1:**
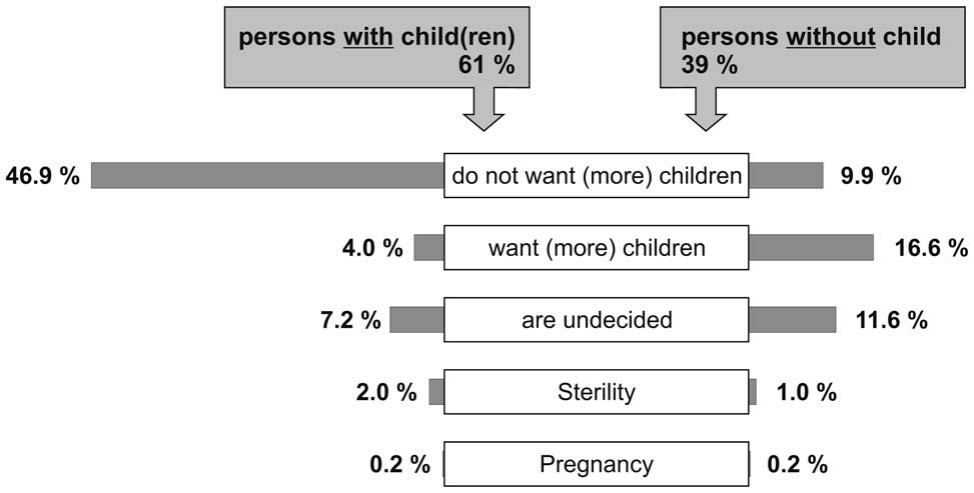
Decision in favour for against having one (more) child.

More than half of respondents (56.9%; N = 1200) reported not desiring to have (more) children at the time the survey was conducted. Further, 19% (N = 398) were undecided on this question. More details are shown in figure 1. Of the total sample, at least 10.0% had been referred for infertility.

### Knowledge About Fertility and Reproductive Medicine

Participants were asked to estimate the age when female fertility gradually starts to decrease. Three percent of participants answered correctly, expecting the fertility decline to start at the age of 25. 24% responded at age 35 and a further 28% of the sample at age 40. One-third responded that female fertility starts to decline at the age of 45 or after menopause (Table 2). Overall, compared to female participants, males estimated that female fertility starts to decrease at a higher age (p = .022). More highly educated people rated the age of female fertility decline more accurately than those less educated (p = .001).

**Table 2:** Knowledge-based questions and results.

		Total N = 2110*	Female	Male	Gender	Education	Education	Education
			N = 1181*	N = 929*	Difference	degree	degree	Difference
					Kendall tau b	Low N = 1638*	High N = 472*	Kendall tau b
At what age does female fertility start to gradually decline?	25 years	64 (3%)	40 (3.4%)	24 (2.6%)	**.022**	29 (1.8%)	35 (7.4%)	**.001**
	30 years	238 (11.3%)	128 (10.8%)	110 (11.8%)		177 (10.8%)	61 (12.9%)	
	35 years	507 (24%)	309 (26.2%)	198 (21.3%)		394 (24.1%)	113 (23.9%)	
	40 years	602 (28.5%)	333 (28.2%)	269 (29%)		470 (28.7%)	132 (28.0%)	
	45 years	388 (18.4%)	209 (17.7%)	179 (19.3%)		312 (19.0%)	76 (16.1%)	
	after menopause	305 (14.5%)	157 (13.3%)	148 (15.9%)		252 (15.4%)	53 (11.2%)	
How many couples do you think remain involuntarily childless in Germany, today?	thru 10%	715 (33.9%)	375 (31.8%)	340 (36.6%)	.111	545 (33.3%)	170 (36.0%)	**.012**
	11–20%	664 (31.5%)	396 (33.5%)	268 (28.8%)		507 (31.0%)	157 (33.3%)	
	21–30%	418 (19.8%)	225 (19.1%)	193 (20.8%)		326 (19.9%)	92 (19.5%)	
	31–50%	227 (10.8%)	132 (11.2%)	95 (10.2%)		193 (11.8%)	34 (7.2%)	
	>50%	45 (2.1%)	29 (2.5%)	16 (1.7%)		39 (2.4%)	6 (1.3%)	
What is your estimate of the likelihood that a woman becomes pregnant following one artificial insemination treatment cycle?	<10%	216 (10.2%)	127 (10.8%)	89 (9.6%)	**.015**	176 (10.7%)	40 (8.5%)	.227
	10–25%	454 (21.5%)	261 (22.1%)	193 (20.8%)		332 (20.3%)	122 (25.8%)	
	26–50%	665 (31.5%)	389 (32.9%)	276 (29.7%)		517 (31.6%)	148 (31.4%)	
	51–80%	573 (27.2%)	305 (25.8%)	268 (28.8%)		461 (28.1%)	112 (23.7%)	
	>80%	157 (7.4%)	75 (6.4%)	82 (8.8%)		121 (7.4%)	36 (7.6%)	
Have you ever heard, seen or readanything about reproductive medicineprior to this study?	yes	1496 (70.9%)	903 (76.5%)	593 (63.8%)	**.001**	1102 (67.3%)	394 (83.5%)	**.001**
	no	440 (20.9%)	193 (16.3%)	247 (26.6%)		390 (23.8%)	50 (10.6%)	
	don’t know	173 (8.2%)	84 (7.1%)	89 (9.6%)		146 (8.9%)	27 (5.7%)	
How would you describe your knowledge about reproductive medicine? −> only participants who answered “yes” to the previous question	N	1496	903	593	**.001**	1102	394	**.002**
	excellent	24 (1.6%)	18 (2.0%)	6 (1.0%)		13 (1.2%)	11 (2.8%)	
	good	189 (12.6%)	131 (14.5%)	58 (9.8%)		137 (12.4%)	52 (13.2%)	
	moderate	672 (44.9%)	446 (49.4%)	226 (38.1%)		476 (43.2%)	196 (49.7%)	
	poor	515 (34.4%)	268 (29.7%)	247 (41.7%)		398 (36.1%)	117 (29.7%)	
	very low or none at all	96 (6.4%)	40 (4.4%)	56 (9.4%)		78 (7.1%)	18 (4.6%)	

* May not add up to 100% due to missing data.

The rate of involuntarily childless couples of all couples in Germany was estimated to be 5 to 10% by more than one third of participants (33.9%), while 31.5% expected 11 to 20% of couples to be involuntary childless, and another 33% of interviewees believed this rate to be higher than 20%. On average it was estimated that 20% of all couples in Germany were involuntary childless. No sex difference was found with regard to this response (p = .111). More highly educated persons supposed the rate of involuntary childless couples lower than less educated people (p = .012).

In-vitro fertilization (IVF) was associated with a success rate of 44% on average by respondents, although 10% estimated a success rate of less than 10% and 21.5% assumed that successful pregnancy following IVF was achieved in 10 to 25% of cases. About one third estimated the success rate as 25 to 50%, and one third of participants estimated it as greater than 50%. Men overestimated the success rate of IVF significantly more often than women (p = .015).

The majority of respondents (70.9%) had already heard, seen or read something about reproductive medicine at the time the survey was conducted. Of this informed sub-sample, their own knowledge about reproductive medicine was self-reported as either excellent or good by 13.3%. 42% of participants rated their knowledge as moderate while more than half reported having poor knowledge. The most frequently used source of information interviewees mentioned was TV (53%), weekly magazines (27%) as well as professional journals or conversation with friends, relatives and acquaintances (21% each). Female as well as more highly educated participants, in comparison to male and less educated respondents, reported having more often heard or read something about reproductive medicine and having better knowledge in this regard.

### Attitudes Towards Assisted Reproductive Medicine

Interest in reproductive medicine was low among participants (M 3.9, with a scale ranging from 1 ‘extremely interested’ to 5 ‘not interested at all’). Of all respondents 5.8% reported being strongly or extremely interested, and nearly one-third had no interest in this topic (Table 3). Male and less educated participants reported significantly lower interest than women and more highly educated persons (p = .001; p = .001). A correlation between the interest in, and the rating of one’s own knowledge of, reproductive medicine was shown (Cramér-V.391; p = .001).

**Table 3:** Attitude-based questions and results.

		Total	Female	Male	Gender	Education	Education	Education
		N = 2110*	N = 1181*	N = 929*	Difference	degree Low	degree High	Difference
					Kendall tau b	N = 1638*	N = 472*	Kendall tau b
How much are you interested inreproductive medicine?	extremely	27 (1.3%)	25 (2.1%)	2 (0.2%)	**.001**	19 (1.2%)	8 (1.7%)	**.001**
	very	94 (4.5%)	57 (4.8%)	37 (4%)		72 (4.4%)	22 (4.7%)	
	moderate	547 (25.9%)	364 (30.8%)	183 (19.7%)		395 (24.1%)	152 (32.2%)	
	little	806 (38.2%)	447 (37.8%)	359 (38.6%)		596 (36.4%)	210 (44.5%)	
	none at all	633 (30%)	286 (24.2%)	347 (37.4%)		555 (33.9%)	78 (16.5%)	
Assuming that you and your partnerdesire children and were not able tohave children the natural way, whatwould you do?	use all poss. of ART	666 (31.6%)	424 (35.9%)	242 (26%)	**.001**	520 (31.7%)	146 (30.9%)	.509
	adopt a child	459 (21.8%)	259 (21.9%)	200 (21.5%)		328 (20.0%)	131 (27.8%)	
	remain childless	468 (22.2%)	224 (19%)	244 (26.3%)		394 (24.1%)	74 (15.7%)	
	don’t know	517 (24.5%)	274 (23.2%)	243 (26.2%)		396 (24.2%)	121 (25.6%)	
Involuntarily childless couplesshould use all possibilities ofreproductive medicine for havingbiological children.	strongly disagree	151 (7.2%)	87 (7.4%)	64 (6.9%)	.565	114 (7.0%)	37 (7.8%)	.414
	somewhat disagree	220 (10.4%)	122 (10.3%)	98 (10.5%)		156 (9.5%)	64 (13.6%)	
	neither agree/disagree	681 (32.3%)	375 (31.8%)	306 (32.9%)		534 (32.6%)	147 (31.1%)	
	somewhat agree	598 (28.3%)	330 (27.9%)	268 (28.8%)		486 (29.7%)	112 (23.7%)	
	strongly agree	454 (21.5%)	264 (22.4%)	190 (20.5%)		342 (20.9%)	112 (23.7%)	
Infertile couples use techniques ofassisted medicine without havingcorrectly calculated treatment risks.	strongly disagree	152 (7.2%)	85 (7.2%)	67 (7.2%)	.274	102 (6.2%)	50 (10.6%)	**.001**
	somewhat disagree	419 (19.9%)	245 (20.7%)	174 (18.7%)		298 (18.2%)	121 (25.6%)	
	neither agree/disagree	870 (41.2%)	487 (41.2%)	383 (41.2%)		692 (42.2%)	178 (37.7%)	
	somewhat agree	472 (22.4%)	251 (21.3%)	221 (23.8%)		388 (23.7%)	84 (17.8%)	
	strongly agree	186 (8.8%)	106 (9%)	80 (8.6%)		148 (9.0%)	38 (8.1%)	
Older women should be able torealize their desire for children byusing reproductive medicine,independent of their age-basedbiological age limitation.	strongly disagree	714 (33.8%)	431 (36.5%)	283 (30.5%)	**.003**	559 (34.1%)	155 (32.8%)	.695
	somewhat disagree	648 (30.7%)	357 (30.2%)	291 (31.3%)		493 (30.1%)	155 (32.8%)	
	neither agree/disagree	444 (21%)	234 (19.8%)	210 (22.6%)		358 (21.9%)	86 (18.2%)	
	somewhat agree	226 (10.7%)	120 (10.2%)	106 (11.4%)		169 (10.3%)	57 (12.1%)	
	strongly agree	71 (3.4%)	35 (3%)	36 (3.9%)		53 (3.2%)	18 (3.8%)	
Application of new techniques of reproductive medicine presents grave ethical conflicts.	strongly disagree	195 (9.2%)	99 (8.4%)	96 (10.3%)	.089	142 (8.7%)	53 (11.2%)	.031
	somewhat disagree	384 (18.2%)	217 (18.4%)	167 (18%)		314 (19.2%)	70 (14.8%)	
	neither agree/disagree	877 (41.6%)	484 (41%)	393 (42.3%)		705 (43.0%)	172 (36.4%)	
	somewhat agree	405 (19.2%)	232 (19.6%)	173 (18.6%)		299 (18.3%)	106 (22.5%)	
	strongly agree	242 (11.5%)	146 (12.4%)	96 (10.3%)		173 (10.6%)	69 (14.6%)	
How would you appraise the advantages and disadvantages of reproductive medicine as a whole?	clear advantages	121 (5.7%)	64 (5.4%)	57 (6.1%)	.423	90 (5.5%)	31 (6.6%)	**.001**
	rather advantages	420 (19.9%)	242 (20.5%)	178 (19.2%)		308 (18.8%)	112 (23.7%)	
	both adv. and disadv.	758 (35.9%)	423 (35.8%)	335 (36.1%)		589 (36.0%)	169 (35.8%)	
	rather disadvantages	352 (16.7%)	207 (17.5%)	145 (15.6%)		266 (16.2%)	86 (18.2%)	
	clear disadvantages	197 (9.3%)	107 (9.1%)	90 (9.7%)		161 (9.8%)	36 (7.6%)	
	undecided	251 (11.9%)	130 (11%)	121 (13%)		216 (13.2%)	35 (7.4%)	

* May not add up to 100% due to missing data.

Participants were also asked questions designed to reveal their values regarding various aspects of reproductive medicine. Asked about what they would do if they desired children but were not able to have them the “natural” way, one third of participants (31.6%) would use all reproductive options. Remaining childless was an option for one in five (22.2%) as was adopting a child (21.8%). More men than women would accept their own childlessness (p = .001).

Approximately half (49.8%) of the participants agreed that childless couples should use all available assisted reproductive techniques for having biological children, while 17.6% disagreed with this statement. Analyses did not reveal differences in sex or education with regard to this opinion (p = .565; p = .414). A third 32.2% assumed that infertile couples used techniques of reproductive medicine without being able to correctly evaluate treatment risks whereas 27.1% did not agree with this supposition. More highly educated people agreed with this statement more often compared to the less-educated (p = .001).

About two-thirds (64.5%) of participants opposed allowing older women to realize their desire for children by using reproductive medicine, independent of their age-based biological limitation, while 14.1% did not. Women were oppose4d to this more often than men (p = .003).

Nearly a third (30.7%) associated ethical conflicts with employing new reproductive medical techniques, while a quarter (27.4%) did not. Ethical conflicts were more often seen by the more highly educated compared to the less-educated (p = .031).

Being asked to generally evaluate advantages and disadvantages of reproductive medicine, 25.6% reported a preponderance of advantages. 26% associated reproductive medicine predominantly with disadvantages, whereas one third (35.9%), saw it as more balanced. A lower educational level was associated with greater indecisiveness towards possible advantages or disadvantages in comparison with higher educational level (p = .001).

## Discussion

This study sought to identify the knowledge and attitudes of the German population towards reproductive medicine and to evaluate any differences in knowledge and attitudes associated with sex or educational level. The authors conclude that reproductive medicine is a subject which is accessible to the German population, though the results indicate some superficiality in knowledge. For example the decline of in female fertility with age was underestimated.

The age-related decline of female fertility was also underestimated in student samples [19], [21]. Bunting & Boivin [24] found that bearing a child early was only seen as a protective factor for fertility by four out of 149 students. In accordance with findings from Rovei et al. [21] the success rate of reproductive medical treatments was clearly overestimated in the present study.

Findings of other studies also indicate that the probability of conceiving after unprotected intercourse was clearly overestimated [19], [25]. As Lampic et al. [19] and Rovei et al. [21] have shown, women’s knowledge about reproductive medicine was better than that of men. Further, more highly educated people more accurately responded about aspects of general reproductive medicine. The combination of deficient knowledge about female fertility and overestimation of the probability of pregnancy occurrence may help to explain why first-time parenthood is being increasingly postponed.

In many cases this deferral might be explained by misinformation suggesting public information that having children can be arbitrarily postponed. So one has to question if accurate concrete knowledge would lead to a modification of behavior concerning the significance of first-time parenthood, if the decision was made earlier. Yet, the clear overestimation of involuntarily childless couples indicates that there is already an awareness of the problem of the unfulfilled wish for children in the German population. This overestimation can be attributed to the perception of increasing childlessness. But often the differentiation between voluntary and involuntary childlessness is not made [26]. The present study did not ask participant’s views regarding the reasons for involuntarily childlessness.

Knowledge gaps about reproductive medicine in the German general population and the associated demand for better education have also been reported elsewhere [20], [21]. The sexual education of young people has previously focused on other aspects, including information about contraceptive methods and preventing sexually transmissible diseases. Certainly, removing the delay of parenthood due to the misunderstanding of age-related decline in female fertility will not be easy. The education of young women about their own fertility done by treating gynecologists was supposed to be an effective approach [21], [27].

In spite of the low interest and state of knowledge regarding the topic of reproductive medicine reported in this study, a broad acceptance of reproductive medical techniques was found. Half the participants stated that childless couples should make use of all reproductive medical techniques to have biological children. In the event of their own involuntarily childlessness, one-third of the respondents were poised to utilize all possibilities provided by reproductive medicine – women far more often than men. Furthermore, the results showed that men were more able to imagine remaining childless than women. An explanation of these findings may be that involuntary childlessness is considered a stressful life event [17], [18], [28], [29]. By the principle of ‘leaving no stone unturned’, reproductive medicine has become a promising solution providing new options for persons concerned. The primary goal of any treatment for infertility should always be to augment spontaneous conception rates [30].

Studies have shown that involuntarily childless couples do not differ from other couples (e.g. mental health), but that childless couples are frequently confronted to explain their situation. This might result in a sense of diminished self-worth as well as a modification of their existing life plan [31]. In contrast to older studies [32], [33] new findings indicate that both men and women suffer from involuntary childlessness [34], [35] including, primarily, psychological impairment (depression and anxiety).

Although reproductive medical techniques are accepted by the German population, one-third believes that couples concerned were not able to correctly assess the risk of utilization. In particular more highly educated people agreed with this statement. Rauprich et al. [36] showed that German couples wishing to have children were not informed about all relevant aspects of reproductive medical techniques to reach a decision for treatment. Findings by Porter et al. [37] demonstrated that most of the couples desiring children were only interested in information that enhanced their chances for having a baby, while disregarding reasons against a treatment. Yet analyses by Stoebel-Richter et al. [17] and Revermann & Hüsing [18] showed that psychosocial counseling within the reproductive medical context is still desirable.

Balancing the pros and cons of reproductive medicine in the present study resulted in a very heterogeneous pattern showing that advantages as well as disadvantages are noticed by the population. As a result of evaluations, 3% of all children born in Germany were produced with the aid of reproductive medicine [38]. By postponing first-time parenthood to a later age due to changing social and economic conditions it is assumed that the user group of reproductive medical treatment will enlarge in the future.

As a critical appraisal of methods, it should be noted that non-standardized, self-designed items were used. According to the cited studies, this is a common practice, but comparability and generalizability of findings were hampered. Due to the cross-sectional study design used, it remained unsolved, the question of whether knowledge and attitudes change in the general population over time remains unanswered.

Findings of this representative study point out that adequate information transfer is needed concerning female fertility as well as success rates and risks of reproductive medicine. Normally, couples get their information about decreasing female fertility if their desire for a child wish is renewed or they are already in fertility treatment. Family planning and reproductive medicine aspects should be an integral part of sex education at school – just like education about contraception and prevention of sexually transmitted diseases. It may be advisable to explore the use of digital and interactive media to reach young people effectively. Other possibilities for the clarification of young women about female fertility and the success rates and risks of reproductive medicine are conversations and the offer of relevant information material during the gynaecological practice. Furthermore population-wide education campaigns should be considered, which can certainly allow an effective information transfer.

At this time, to inform patients’ effective balancing of the success rates and risks of reproductive medical techniques in terms of patient-centered treatment decision-making, broad education and counseling both at a medical and psychosocial level are necessary to address current knowledge deficiencies and attitudes of the general population.
